# Progressive Brain Degeneration From Subjective Cognitive Decline to Amnestic Mild Cognitive Impairment: Evidence From Large-Scale Anatomical Connection Classification Analysis

**DOI:** 10.3389/fnagi.2021.687530

**Published:** 2021-07-12

**Authors:** Wuhai Tao, Hehui Li, Xin Li, Rong Huang, Wen Shao, Qing Guan, Zhanjun Zhang

**Affiliations:** ^1^Center for Brain Disorders and Cognitive Science, Shenzhen University, Shenzhen, China; ^2^Shenzhen-Hong Kong Institute of Brain Science-Shenzhen Fundamental Research Institutions, Shenzhen, China; ^3^State Key Laboratory of Cognitive Neuroscience and Learning and IDG/McGovern Institute for Brain Research, Beijing Normal University, Beijing, China; ^4^Department of Neurology, China-Japan Friendship Hospital, Beijing, China

**Keywords:** amnestic mild cognitive impairment, subjective cognitive decline, white matter, network, Alzheimer’s disease

## Abstract

People with subjective cognitive decline (SCD) and amnestic mild cognitive impairment (aMCI) are both at high risk for Alzheimer’s disease (AD). Behaviorally, both SCD and aMCI have subjective reports of cognitive decline, but the latter suffers a more severe objective cognitive impairment than the former. However, it remains unclear how the brain develops from SCD to aMCI. In the current study, we aimed to investigate the topological characteristics of the white matter (WM) network that can successfully identify individuals with SCD or aMCI from healthy control (HC) and to describe the relationship of pathological changes between these two stages. To this end, three groups were recruited, including 22 SCD, 22 aMCI, and 22 healthy control (HC) subjects. We constructed WM network for each subject and compared large-scale topological organization between groups at both network and nodal levels. At the network level, the combined network indexes had the best performance in discriminating aMCI from HC. However, no indexes at the network level can significantly identify SCD from HC. These results suggested that aMCI but not SCD was associated with anatomical impairments at the network level. At the nodal level, we found that the short-path length can best differentiate between aMCI and HC subjects, whereas the global efficiency has the best performance in differentiating between SCD and HC subjects, suggesting that both SCD and aMCI had significant functional integration alteration compared to HC subjects. These results converged on the idea that the neural degeneration from SCD to aMCI follows a gradual process, from abnormalities at the nodal level to those at both nodal and network levels.

## Introduction

The current status of Alzheimer’s disease (AD) clinical treatment is not promising, which makes preclinical prediction for AD particularly important (Huang et al., 2020). Many studies have shown that AD manifests significant pathological changes decades before it develops into dementia (Jack et al., 2010; Bateman et al., 2012). Characterized by objective cognitive impairment similar to AD, mild cognitive impairment has been proposed as an important stage in the development of AD. In particular, about one-third of those with amnesiac mild cognitive impairment (aMCI) will develop AD within 5 years (Ward et al., 2013). Similarly, the elderly with subjective cognitive decline (SCD) also has a high risk for developing AD (Jessen et al., 2020). Both at the early stages of AD, the major behavioral difference between SCD and aMCI is that aMCI has severer objective cognitive impairments than SCD. However, knowledge about the relationship between SCD and aMCI neuroimaging characteristics is still insufficient.

Some studies have found that aMCI and SCD have similar structural or functional degeneration with AD (Scheef et al., 2012; Wang et al., 2013, 2016). In general, patients with aMCI had more extensive and severe neurological impairments than the elderly with SCD (Sun et al., 2015). However, regions of differences in structural and functional activities between aMCI and SCD are not the same in different studies. For example, SCD may, in some way, compensate for the negative effects of neurological damage in some distracted areas to ensure that they have normal performance when completing cognitive ability tests (Erk et al., 2011). Recently, studies have shown that neural impairments of aMCI and SCD are not only restricted to individual regions but also extended to the interactions among multiple brain areas (Dai and He, 2014; Tao et al., 2020). Consistent with this, in the last 5 years, extensive research has been conducted on neural substances associated with AD and its development from the perspective of brain networks (Wang et al., 2016; Shu et al., 2018; Lazarou et al., 2019). Graph theoretical analysis offered a new perspective to estimate the changes of multiple properties of brain networks, both at the local and global level, as the disease progresses (Bullmore and Sporns, 2009; He and Evans, 2010). There were also some researchers who suggested that brain connectome research provided a very effective way for SCD studies (Lazarou et al., 2019).

Functional segregation, which can reflect the local information processing, and functional integration, which is a reflection of the global information processing, are two major aspects of the information activity of the brain. In the graph theoretical analysis, the index of clustering coefficient and local efficiency, global efficiency, and path length of brain networks can effectively reflect the two aspects, respectively (Rubinov and Sporns, 2010). One previous study has shown that AD patients had lower brain network integration and higher brain network segregation, and these changes were significantly correlated with cognitive decline (Kabbara et al., 2018). The combination of the features of brain network integration and segregation can distinguish AD patients from healthy elderly with high accuracy (Cai et al., 2020). An earlier review article on the topic of structural and functional networks in the brain reported both functional segregation and functional integration impairments in MCI and AD (Dai and He, 2014). Our previous work also revealed impairments of anterior–posterior brain functional connectivity in SCD in the resting state (Tao et al., 2020). These results may indicate that the brain network of SCD has also been altered. In the meantime, considering that the pathological changes of AD are a gradual process, it is suggested that the problems with the integration and separation of brain networks might occur at both aMCI and SCD stages.

To be considered as a disconnection syndrome (Delbeuck et al., 2003), the white matter (WM) connectivity plays a crucial role in the progress of AD pathology. The microstructural deterioration of WM caused by demyelination and axonal deterioration may result in obstacles of information transfer within the brain network (Bozzali et al., 2002). Both aMCI and SCD have been reported with widespread WM impairment in previous studies (Selnes et al., 2013; Defrancesco et al., 2014; Shao et al., 2019), which further disrupted the topological properties of their brain network (Wang et al., 2016; Shu et al., 2018). Moreover, the degree of WM abnormalities is significantly correlated with the neurofibrillary tangle pathology stage and the severity of the disease (Kantarci et al., 2017). Since aMCI and SCD are in different stages of AD, it is necessary to look further into the phase-specific characteristics of aMCI and SCD WM networks to clarify the structural basis of the specific behavior in each stage.

To address this issue, in the current study, diffusion tensor imaging and deterministic tractography were first used to construct the WM structural network. We then used graph theory approaches to estimate neural indexes, including path length, the global efficiency, the local efficiency, and the clustering coefficient, both the nodal and network levels. Finally, classification models were built to investigate which indexes can significantly identify SCD or aMCI from HC. We hypothesized that neural degeneration follows a gradual change from SCD to aMCI. Specifically, neural differences between SCD and HC were mainly represented by indexes at the node level, whereas that between aMCI and HC were represented by indexes at the network level. We also examined the functional segregation and integration properties between SCD or aMCI and HC, and made further assumption that SCD and aMCI are already impaired in both, but given that they are at distinct stages, there may be subtle differences in the manifestation of the neural impairments between the two.

## Materials and Methods

### Participants

A total of 66 participants (mean age, 64.76 ± 6.4) were recruited in the current study, including 22 SCD, 24 aMCI, and 23 gender-, age-, and years of education-matched HC. All participants were sourced from the Beijing Aging Brain Rejuvenation Initiative database, which is a project of community-based elderly health study. Participants meeting the following criteria were included: (1) having no less than 6 years of education and being able to complete a series of neuropsychological measurements; (2) nondementia, the score of Mini-Mental Status Examination (MMSE, Chinese version) ≥ 24; (3) no history of coronary disease, psychotic disorders, tumors, motor neuron disease, developmental disability, or diseases that could influence cerebral function; (4) no clinical diagnosis of depression, schizophrenia, and other psychiatric disorders, and no history of taking psychoactive medications; and (5) no physical problems that are not appropriate for MRI scan.

In addition to the above criteria, the SCD participants also had (1) self-reported memory declines in recent years relative to previous states but was not caused by acute events, (2) the normal cognitive function above -1.5 standard deviations (SD) of the Chinese norms, and (3) intact daily living function. For the aMCI participants, they should meet the published inclusion criteria by Petersen et al. (1999): (1) had memory declined complaints, (2) scores of cognitive function below 1.5 SD of the Chinese norm, and (3) no difficulty in daily life.

### Neuropsychological Assessment

A series of neuropsychological assessments were used to assess the general mental status and other cognitive functions of all the participants. The general cognitive function was measured by the MMSE, while the memory function was estimated by the Auditory Verbal Learning Test (AVLT) and the Rey-Osterrieth Complex Figure test (ROCF-recall). The Symbol Digit Modalities Test (SDMT) and part A of the Trail Making Test (TMTa) tested the attention ability, while the part B of the TMT (TMTb) and part C of the Stroop Test (Stroop C) tested the executive function.

### Image Acquisition

A Siemens 3.0T scanner (Siemens, Munich, Germany) was employed to acquire the MRI imaging data at the Imaging Center for Brain Research, Beijing Normal University. Participants lay flat on their backs with foam pads to minimize head motion. T1-weighted images were acquired using sagittal 3D magnetization prepared rapid gradient echo (MP-RAGE) sequences. The acquisition parameters were as follows: repetition time (TR) = 1,900 ms, echo time (TE) = 3.44 ms, flip angle = 9°, field of view (FOV) = 256 × 256 mm^2^, and acquisition matrix = 256 × 256, 1 mm slice thickness, and 176 sagittal slices. Diffusion-weighted images were obtained by an echo-planar imaging sequence with the parameters as follows: TR = 11,000 ms; TE = 94 ms; flip angle = 90°, FOV = 240 × 240 mm^2^, acquisition matrix = 128 × 128, 2 mm slice thickness, and 70 sagittal slices. The diffusion sensitizing gradients were applied, 1 image without diffusion-weighted (*b* = 0 s/mm^2^) and 30 diffusion-weighted directions (*b* = 1,000 s/mm^2^).

### Image Preprocessing

MATLAB 2018a, SPM12^1^, and PANDA (Pipeline for Analysing Brain Diffusion Images)^2^ software were used to preprocess the DTI images. Several steps were applied to the data preprocessing: eddy current and motion artifact correction, fractional anisotropy calculation, whole-brain fiber tracking, and diffusion tensor tractography. The fiber tracking was performed by the continuous tracking algorithm, and the fiber tracts were terminated if two consecutive moving directions have a crossing angle above 45°and the fractional anisotropy is out of the threshold 0.2–1 (Cui et al., 2013).

### Network Construction

**FIGURE 1:**
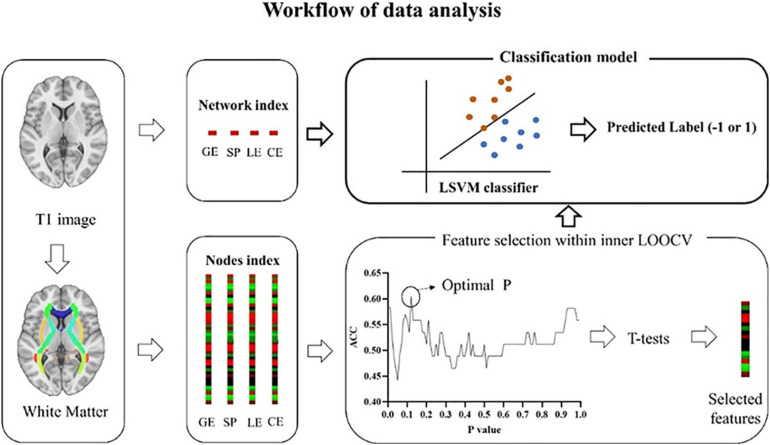
Workflow of data analysis. White matter matrices were constructed based on the AAL template, an automated anatomical parcellation of the spatially normalized single-subject high-resolution T1 volume, during which indexes (GE, SP, LE, and CE) at the network and nodal level were extracted. At the network level, four indexes, including GE, SP, LE, and CE were used separately or combined to build a classifier. GE, global efficiency; SP, short path length; LE, local efficiency; CE, clustering coefficient. At the nodal level, each index includes 90 features. To select the most discriminate features, LSVM (an outer LOOCV) was nested with a feature selection produce (an inner LOOCV). LSVM, linear support vector model; LOOCV, leave-one-out cross-validation. Similar to nodal level, four indexes were used separately or combined to build a classifier.

Network nodes and edges are the most basic element of a brain network. We used the standard procedure proposed by Gong et al. (2009) and constructed the WM network as Shu et al. (2018) described in their work. Network nodes were defined using the 90 brain regions subdivided by the automated anatomical labeling (AAL) template. The network nodes were considered structurally connected if the number of fibers between two nodes was ≥3 (Shu et al., 2012). We set the thresholds to 1–5 and 10 and observed the effects of diverse thresholds on the differential characteristics between groups, respectively, and no significant changes were observed between the thresholds (see Figure 1 and Supplementary Table 1). Then, the number of valid fibers (FN) between regions was defined as the weights of the network edges. Eventually, each subject was constructed an FN-weighted 90 × 90 matrix WM network.

### Network Analysis

For each subject, the GRETNA software^3^ was applied to quantify the network metric. The characteristic path length and the global efficiency, which can reflect the structure integration, the local efficiency, and the clustering coefficient, indicating the structure segregation, were calculated for each participant at both network and nodal levels. The smaller the characteristic path length and the higher the local efficiency, the better the structure integration. The higher the local efficiency and the higher the clustering coefficient, the better the structure segregation. The BrainNet Viewer^4^ was used to present the network results.

#### Indexes at the Network Level

The characteristic path length described the mean of the shortest path length between nodes. It can be computed as follows:

$$L\,\left(G\right)=\frac{1}{N\,\left(N-1\right)}\,\sum_{i\neq j\in G}d_{i\,j}$$

The global efficiency measures the efficiency of parallel information transfer in the network. It can be computed as follows:

$$E_{\mathrm{global}}\,\left(G\right)=\frac{1}{N\,\left(N-1\right)}\,\sum_{i\neq j\in G}\frac{1}{d_{i\,j}}$$

The local efficiency shows how efficient the communication is among the neighbors of each node. It can be computed as follows:

$$E_{\mathrm{loc}}\,\left(G\right)=\frac{1}{N}\,\sum_{i\in G}E_{g\,l\,o\,b}\,\left(G_{i}\right)$$

The clustering coefficient is defined as the possibility of the neighborhoods that are connected with each other. It can be computed as follows:

$$C\,\left(G\right)=\frac{1}{\text{N}}\,\sum_{i\in G}C\,i=\frac{1}{N}\,\sum_{i\in G}\frac{2\,{\text{t}}_{i}}{k_{i}\,\left(k_{i}-1\right)}$$

#### Indexes at the Nodal Level

The shortest path length of node i shows the mean distance between node i and other nodes. It can be computed as follows:

$$L\left(i\right)=\max_{j}d\left(1\to j\right)$$

The nodal efficiency for node i shows the efficiency of parallel information transfer of this node in network G. It can be computed as follows:

$$E_{g\,l\,o\,b}\,\left(i\right)=\frac{1}{N-1}\,\sum_{j\neq i\in G}\frac{1}{d_{i\,j}}$$

The local efficiency of node i shows the efficiency of the communication among the first neighbors of node i when it is removed. It can be computed as follows:

$$E_{l\,o\,c}\,\left(i\right)=\frac{1}{N_{i}\,\left(N_{i}-1\right)}\,\sum_{j\neq i\in G_{i}}\frac{1}{d_{i\,j}}$$

The clustering coefficient of node i shows the likelihood the neighbors of node i connected to each other. It can be computed as follows:

$$\text{C}\,\left(\text{i}\right)=\frac{2\,t_{i}}{k_{i}\,\left(k_{i}-1\right)}$$

In all the formulas above, N is the total number of nodes in the network, d_*ij*_ is the shortest path length between node i and node j in network G, and G_*i*_ denotes the subgraphs of node i. C_*i*_ represents the clustering coefficient of node i, N is the total number of nodes, t is the weighted edges, and k is the number of nodes connecting to node i.

### Statistical Analysis

General statistical analyses were performed with SPSS (version 22.0, Chicago, IL, United States). Analysis of covariance (ANCOVA) with age, gender, and years of education as covariates was used to estimate the group difference in neuropsychological tests, global network metrics, and nodal properties. If the main effects of groups were significant, *post hoc t*-tests were performed to further examine the difference between any two groups. A false discovery rate (FDR) correction was performed at a *q*-Value of 0.05 to correct for multiple comparisons. Receiver operating characteristic curve (ROC) analysis was applied to describe the discrimination of network and nodal characteristics on HC, SCD, and aMCI. We also did partial correlation analysis with age, gender, and years of education adjusted to reveal the relationship between cognition and some WM indicators that we selected.

### The LSVM-Based Classification

An LSVM method was performed using LIBSVM for Matlab^5^ (Chang and Lin, 2011) to differentiate aMCI or SCD from HC with WMV metrics. The leave-one-out cross-validation (LOOCV) was applied for the cross-validation, which has been widely used in previous studies, especially for data with a small sample size (Cui et al., 2016; Pereira et al., 2009). In this dataset, multiple dimensional spaces were represented by all of the features, and each participant was a point in these multiple dimensional spaces. The LSVM used a subset of data (i.e., training set, n - 1 participants) as input to build a modal that can best separate the input data into two categories (Cui et al., 2016). Then, a relatively dependent dataset was used (i.e., testing set, the last participant) to test this classifier. Based on the features that have been used, the last participant can be classified as any of the two classes (e.g., HC or SCD), labeled as 1 or -1. If the predicted label is consistent with the real label, then the classification is correct. After the leave-one-out loop for two groups was finished, a final accuracy represented by the probability to predict accurately can be calculated, which demonstrated the performance of the classification model.

### Feature Selection at the Nodal Level

Each index (global efficiency, local efficiency, clustering efficiency, and short path) at the node level was represented by a 90 × 1 matrix. To boost classification performance, a nested inner LOOCV loop was conducted, with the outer loop to estimate classification accuracy and the inner loop to select discriminative features and to eliminate the noninformative features (Cui et al., 2016). Detailed steps were as follows. First, N - 1 subjects were used as the training set, the last participant served as the testing set for the outer LOOCV loop. Second, all data were normalized (Cui et al., 2016). Third, an inner LOOCV loop was performed, during which two-sample *t*-tests were applied for each feature within N - 2 participants. Features below a given *p*-Value were selected for inner classification models. The given *p*-Value was set from 0.01 to 0.99 with a step of 0.01. In this way, 99 inter-LOOCV were conducted, and 99 accuracies were obtained. Optimal *p* was determined by the highest classification accuracy. (4) Features thresholded with this optimal *p*-Value were selected for the training set of the outer LOOCV loop. (5) The resultant discriminative weight for each feature was calculated to mark the relative importance of a feature to a classifier (Mourao-Miranda et al., 2005). Notably, this strict feature selection procedure was skipped for indexes at the network level.

#### Definition of the Discriminate Features

Features selected for each outer loop were slightly different because of the difference in the dataset (n - 1 participants for each time). The absolute weight of features that were used for all outer loops was averaged, which was used to indicate the discriminate weight of each feature (Dai et al., 2012; Cui et al., 2016, Cui and Gong, 2018). The higher the discriminate weight is, the greater the contribution of the corresponding feature to the classifier is. In the current study, the most discriminate features were defined as those with averaged discriminate weight larger than 0.

### Evaluation of Classification Performance

Accuracy, specificity, sensitivity, area under the ROC curve (AUC) were estimated to quantify the classification accuracy. As presented earlier, accuracy means the proportion of subjects to be accurately classified. Specificity means the proportion of subjects who can be accurately classified as HC or SCD (or aMCI). Sensitivity means the proportion of subjects who can be accurately classified as SCD (or aMCI). Furthermore, ROC analysis was used to estimate the effectiveness of each classifier. The AUC indicates the classification performance of a classifier, and a larger AUC represents a better performance (Fawcett, 2006). To estimate the significance of the accuracy and AUC, a 1,000 permutation test was performed to build null distributions, during which labels for each participant were shuffled and the whole classification procedure was reperformed.

## Results

### Demographic Information

Age, gender, and years of education did not differ significantly among the three groups. MMSE was significantly higher in HC than in SCD and aMCI. Memory and executive functions declined in the order of HC, SCD, and aMCI. The attentional function was significantly stronger in both HC and SCD than in aMCI (Table 1). We have reported these behavioral results in another article as well (Tao et al., 2020).

**TABLE 1:** Demographics and neuropsychological characterizations.

	**HC (*n* = 23)**	**SCD (*n* = 22)**	**aMCI (*n* = 24)**	**χ ^2^/F**	***p***
Gender (m/f)	14/9	11/11	11/13	1.13	0.570
Age (years)	65.91 ± 5.86	62.41 ± 5.24	65.71 ± 7.49	2.19	0.120
Education (years)	12.30 ± 2.84	10.50 ± 2.82	10.50 ± 2.96	3.02	0.056
MMSE	29.04 ± 0.93	27.14 ± 1.73	26.04 ± 1.78	21.08^a^	<0.001
Memory	6.05 ± 0.40	4.94 ± 0.40	4.04 ± 0.37	151.96^b^	<0.001
Attention	5.58 ± 0.48	5.35 ± 0.57	4.12 ± 0.83	29.79^c^	<0.001
Executive	5.61 ± 0.27	5.11 ± 0.43	4.31 ± 0.84	30.64^b^	<0.001

*Values are mean ± standard deviation. All covariance analyses used gender, age, and education as covariables. The *post hoc* tests were corrected by Bonferroni, and *p* < 0.05 was considered significant. ^*a*^HC > SCD and HC > aMCI. ^*b*^HC > SCD > aMCI. ^*c*^HC > aMCI and SCD > aMCI.*

### The Mean Structural Network Matrix

**FIGURE 2:**
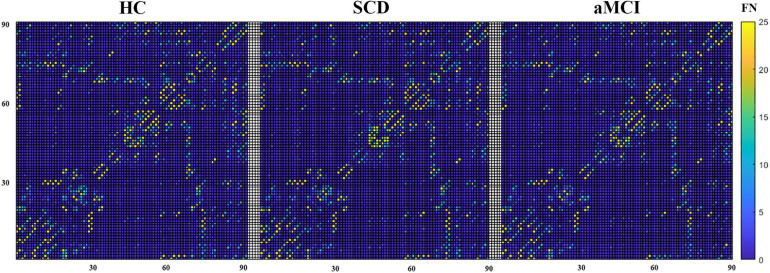
The mean structural network matrix of each group built on the white matter. The horizontal and vertical coordinates represent 90 brain regions in the AAL template. Values in each cell represent the mean FN between two brain areas.

The mean structural network matrix for the three groups is presented in Figure 2. Notably, the number of fiber tracts between a few brain regions was abundant (nearly 300), but we chose only 25 as the maximum threshold to achieve a better representation of the structural connectivity state between most brain regions. A two-tailed t-test for group comparisons of the structural network (p < 0.025) was presented in the Supplementary Material. Most group differences were found between HC and aMCI, while relatively few differences were found between HC and SCD (see Supplementary Figure 2).

### Classification With White Matter Indexes at the Network Level

**FIGURE 3:**
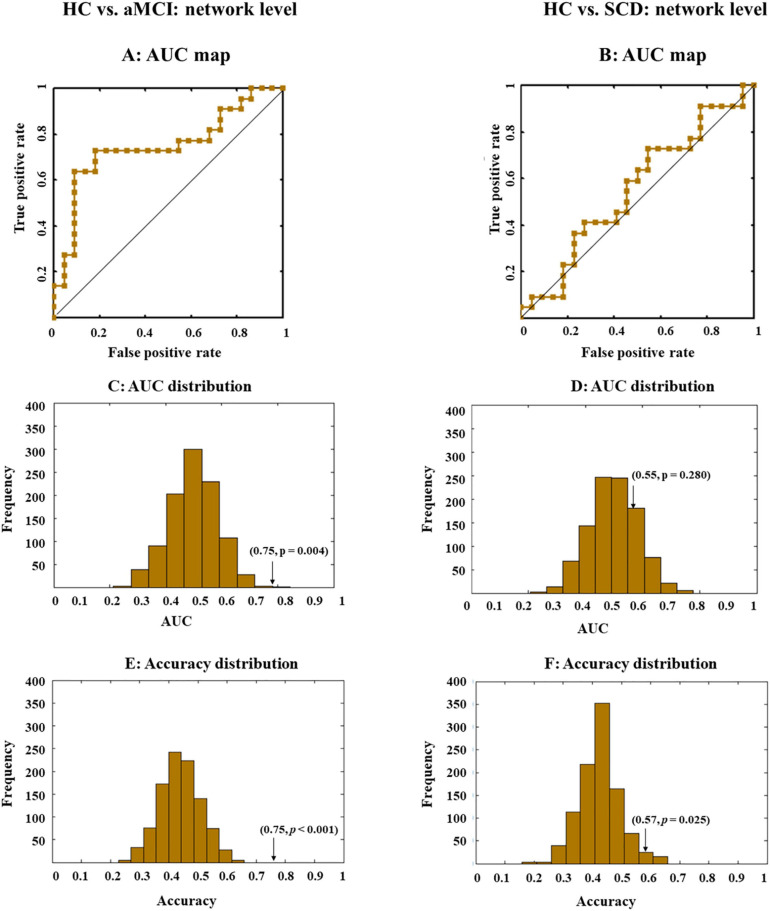
Receiver operating characteristic (ROC) and area under the curve (AUC) or accuracy distribution when all indexes (i.e., global efficiency, local efficiency, cluster efficiency, and short path) at the network level were combined. ROC maps shows the classification performance **(A,B)**, where a greater AUC corresponds to better performance. AUC and accuracy distribution maps **(C–F)** were built by permutation tests, during which group labels were randomly arranged 1,000 times. Arrows in the distribution maps marker AUC or accuracy based on real group labels.

The LSVM classifier accurately discriminated aMCI from HC when all network indexes (i.e., global efficiency, local efficiency, cluster efficiency, and short path) were combined (brown lines in Figure 3A). The result elucidated significant AUC (0.75, *p* < 0.001; Figure 3E and Table 2) and accuracy (0.75, *p* < 0.001; Figure 3C and Table 2, bold font), respectively. Then, LSVM was also conducted by using the global efficiency, local efficiency, clustering efficiency, and short path separately. Permutation tests revealed significantly higher accuracy and AUC for all indexes except for the clustering coefficiency (Table 2).

**TABLE 2:** Clustering performance based on each index.

	**HC vs. aMCI**				**HC vs. SCD**			
**Network level**								
	**Accuracy**	**AUC**	**Sensitivity**	**Specificity**	**Accuracy**	**AUC**	**Sensitivity**	**Specificity**
**Combined**	**0.75**	**0.75**	**0.59**	**0.90**	0.57	0.55	0.64	0.50
Global efficiency	0.68	0.77	0.59	0.77	0.45	0.34	0.73	0.18
Local efficiency	0.68	0.71	0.59	0.77	0.55	0.49	0.73	0.36
Clustering co-efficiency	0.16	0.09	0.18	0.14	0.50	0.52	0.59	0.41
Path length	0.70	0.76	0.55	0.84	0.39	0.41	0.36	0.41
**Nodal level**								
Combined	0.52	0.52	0.50	0.55	0.43	0.39	0.41	0.45
Global efficiency	0.55	0.57	0.50	0.59	**0.73**	**0.71**	**0.68**	**0.77**
Local efficiency	0.61	0.63	0.64	0.59	0.32	0.29	0.41	0.23
Clustering co-efficiency	0.45	0.48	0.41	0.50	0.55	0.61	0.50	0.59
Path length	**0.66**	**0.66**	**0.59**	**0.73**	0.64	0.57	0.50	0.77

However, for SCD and HC, performance accuracies for all classifiers either building on each index or the combined index were all lower than 0.6, with nonsignificant AUCs close to 0.5 (Figures 3B,D,F).

### Classification With White Matter Indexes at the Nodal Level

**FIGURE 4:**
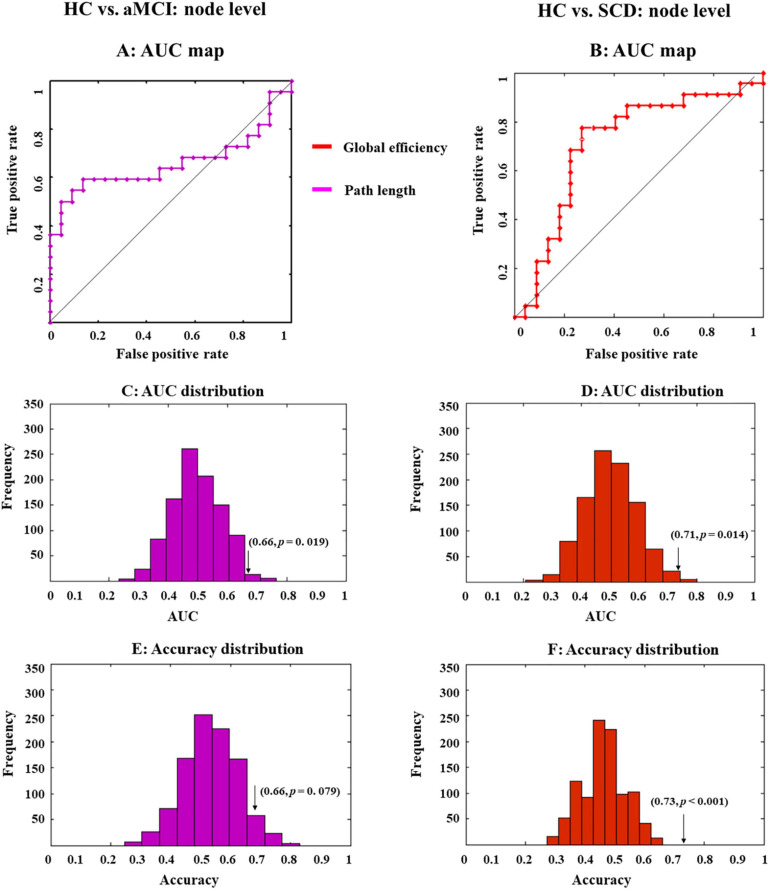
Receiver operating characteristic (ROC) maps and area under the curve (AUC) or accuracy distribution of a classifier built on indexes at the nodal level. ROC maps show the classification performance **(A,B)**, where greater AUC corresponds to better performance. AUC and accuracy distribution maps **(C–F)** were built by permutation tests, during which group labels were randomly arranged 1,000 times. Arrows in the distribution maps marker AUC or accuracy based on real group labels.

Then, the LSVM was conducted at the node level. We first combined all indexes to classify different groups. However, the whole-brain anatomical connection pattern cannot significantly separate aMCI or SCD from HC (Figures 4A,B).

Then, we exploited single-type metrics to build classifiers, during which observed a double-dissociation pattern. Specifically, path length can differentiate aMCI and HC but not SCD and HC (Figure 4A). Notably, AUC for this classifier was significant (0.66, *p* = 0.019; Figure 4C and Table 2, bold font), while accuracy was marginally significant (0.66, *p* = 0.079; Figure 4E and Table 2). Even though the significance was marginal, there was still a trend that path length might be able to identify aMCI from HC. In addition, the most 10 discriminative WM features for this classifier were the left supramarginal gyrus; left amygdala; right inferior frontal gyrus, opercular part; right hippocampus; left temporal pole; middle temporal gyrus; right superior temporal gyrus; left inferior frontal gyrus, triangular part; left lenticular nucleus, pallidum; right inferior parietal, but supramarginal and angular gyri; and right thalamus.

On the other hand, the global efficiency can significantly identify SCD but not aMCI from HC (Figure 3). AUC (0.71, *p* = 0.014; Figure 4D and Table 2, bold font) and accuracy (0.73, *p* < 0.001; Figure 4F and Table 2) were all significant, with most discriminative WM features consisting of the left lenticular nucleus, pallidum; right fusiform gyrus; and right lenticular nucleus, pallidum.

### Correlation Between Network Metrics and Cognition

**FIGURE 5:**
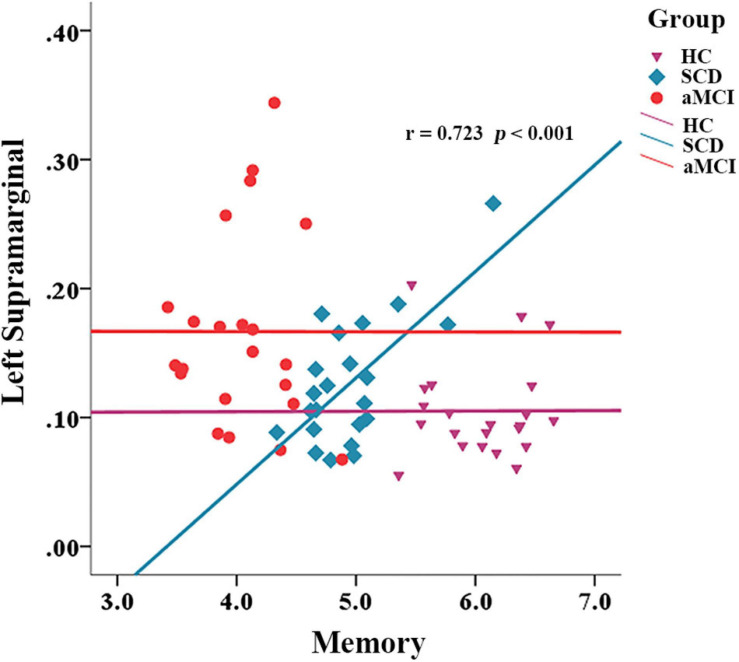
Correlation between network metrics and memory in different groups.

We also calculated the correlation between network properties and cognition with gender, age, and years of education as covariates in each group. In the SCD group, the path length of the left supramarginal was positively correlated with memory ability (*r* = 0.723, *p* < 0.001), but this was not the situation in the group of HC and aMCI (Figure 5).

## Discussion

We investigated the topological characteristics of the WM network that can successfully identify SCD and aMCI from HC. Compared to the HC group, the combination of multiple indexes at the network level was able to significantly distinguish aMCI, but not SCD. The classifiers built on different indexes at the nodal level can identify SCD and aMCI from HC. Specifically, the short-path length at the nodal level was able to significantly distinguish aMCI from HC, whereas the global efficiency at the nodal level was able to identify SCD from HC. The most discriminative short-path length features include the left supramarginal, the left amygdala, the opercularis part of the right inferior frontal, the right hippocampus, and the left temporal pole, whereas the most discriminative global efficiency features contain the left pallidum, the right fusiform, and the right pallidum. Besides, we found that the short-path length of the left supramarginal was significantly correlated with memory performance in the SCD group.

Abnormalities in white matters, which were responsible for transmitting information between brain regions, often cause impairments of higher cognitive functions requiring the collaboration of multiple brain regions. The impairments of WM in AD have been reported in previous studies, which have observed that WM impairments are significantly associated with cognitive decline in AD (Overdorp et al., 2014; Bilello et al., 2015). WM damages are one of the typical features of the pathological development of AD (Lee et al., 2016). This WM degeneration, representing demyelination and microhemorrhages, is also frequently reported in aMCI and SCD (Ohlhauser et al., 2019; Bangen et al., 2020; Li et al., 2020). In the current study, we focused on structural properties (white matter metric) of the brain; therefore, we selected the AAL template that was built on high-resolution T1 volume and has been widely used in previous brain network research (Feng et al., 2015; Qi et al., 2015; Zhuo et al., 2018). Consistent with other studies (Wang et al., 2016; Shu et al., 2018), disconnection symptoms and dynamic network failure in AD were found to be of great significance in the preclinical stages of AD, and this provides the neurostructural basis for the altered behavioral manifestations in these stages.

Compared to aMCI, SCD is at a much earlier stage, where the individual’s cognitive abilities are still relatively intact. Our findings also reveal the stage-specific characteristics of WM network disruption in each group. Both aMCI and SCD exhibit impairments in network topological properties at the nodal level, but indexes at the network level can only significantly distinguish aMCI from HC, with no significant classification power in discriminating between SCD and HC. This suggests a progressive degeneration of whiter matter from SCD to aMCI, from WM impairments at the local level to both local and global levels. There is extensive evidence that SCD has similar WM degeneration to aMCI and AD (Selnes et al., 2012; Li et al., 2016), and the degree of degeneration is often intermediate between HC and aMCI (López-Sanz et al., 2017). Our current study further shows that the WM network damage in SCD has not reached the overall level of the whole brain network as in aMCI but is only limited to some local nodes. In this case, SCD may also be able to compensate for the problems caused by local node degeneration through resource allocation at the overall network level or nodes that has been impaired, just like the alternative enhancement of partial regional activation reported by previous authors in functional MRI (Erk et al., 2011). This may be one of the reasons why SCD is still able to maintain good performance in cognitive tasks.

Based on the nodal-level analysis, we found that the attributes that best distinguish SCD and aMCI from HC are the global efficiency and the short-path length. These are two indicators representing the extent of function integration among brain regions (Bullmore and Sporns, 2012). The results suggest that both SCD and aMCI might have pronounced functional integration issues. They may have much more difficulties in accomplishing those higher cognitive functions that require multiple brain regions to collaborate together. Several previous studies have reported altered functional integration and functional segregation in both preclinical stages of AD (Lazarou et al., 2019), and some investigators have suggested that enhanced functional segregation compensates for the problems associated with functional integration impairments (Xu et al., 2020), but the latter was not as evident in our study. In addition, those elderly with amyloidosis are more likely to have functional integration problems (Fischer et al., 2015). This further confirms that SCD and aMCI are two important preclinical risk stages for AD.

We used the fiber number of WM to build the network. The change in the short-path length in aMCI suggests that some of the WM pathways between the nodes have been impaired. The changes in the global efficiency of some nodes in SCD also indicate that the efficiency of information exchange between these nodes and other regions of the brain has been impaired. Some of the nodes with the greatest discriminatory validity were identified in our analysis, and these nodes were mainly distributed in temporal and frontal regions, which is consistent with the sequence of pathological development in early AD (Serrano-Pozo et al., 2011). We found a significant correlation between the left marginal superior gyrus and memory in the SCD group. Some studies suggest that thinner volumes in this region are associated with an increased risk of AD (Verfaillie et al., 2016). As part of the attentional network (Yeo et al., 2011), the marginal supramarginal gyrus has shown an important role in memory encoding in several studies (Staresina and Davachi, 2006; Rubinstein et al., 2021). In addition, the literature shows that supramarginal gyrus is often functionally involved in action execution, simulation, and observation (Grezes and Decety, 2001), and some researchers have suggested that activation of this component when subjects are memorizing items may reflect the action-oriented approach to memory adopted by the subjects (Russ et al., 2003). However, somewhat curiously, we found that the mean path length of the left marginal superior gyrus, in relation to other nodes of the whole brain, showed a significant positive correlation with memory performance in the SCD group. It appears that better structural segregation of this region from other nodes contributes to better memory performance. In fact, it can be observed from the scatter plot (Figure 4) that the HC group did also have a longer mean path length than the aMCI group. We speculate that perhaps the region is undergoing a transition from a cost-effective network to a random network during the SCD stage and that some unnecessary connections to this brain region may cause interference with memory function. This is consistent with the phenomenon of brain dedifferentiation in aging and disease development (Goh, 2011; Caldwell et al., 2020). This is still only our conjecture, and follow-up studies need more experimental evidence to further validate it.

Some limitations of our study should be mentioned. First, our study sample is relatively small, which may affect the generalizability of our current findings, and the seniors who participated in the current study were from the community. While we believe in the importance of focusing on the community elderly, SCD from the clinic did have a higher AD conversion rate (Jessen et al., 2020). Second, the current study is only a cross-sectional study, and the findings need to be confirmed by future longitudinal studies. Third, combining other neuroimaging features such as cerebrospinal fluid markers with the WM network alterations in the current study will help to reveal a more comprehensive picture of the preclinical pathological changes in AD. In conclusion, our study shows that both SCD and aMCI have impairments in the functional integration of WM networks relative to HC and that network impairments in aMCI have undergone a quantitative change from the nodal level to the network level.

## Data Availability Statement

The datasets presented in this article are not readily available because raw data will be made public upon completion of subsequent studies. Requests to access the datasets should be directed to twh621@foxmail.com.

## Ethics Statement

The studies involving human participants were reviewed and approved by the Institutional Review Board of the Beijing Normal University Imaging Centre for Brain Research, China, Approval No. ICBIR_A_0041_002_02. The patients/participants provided their written informed consent to participate in this study.

## Author Contributions

WT contributed to the conceptualization, investigated the data, carried out the formal analysis and funding acquisition, wrote the original draft, and reviewed and edited the manuscript. HL carried out the formal analysis and funding acquisition, wrote, reviewed, and edited the manuscript. XL wrote, reviewed, and edited the manuscript. RH and WS investigated the data and wrote, reviewed, and edited the manuscript. QG and ZZ contributed to the conceptualization, wrote, reviewed, and edited the manuscript, carried out the project administration and funding acquisition, and supervised the data. All authors contributed to the article and approved the submitted version.

## Conflict of Interest

The authors declare that the research was conducted in the absence of any commercial or financial relationships that could be construed as a potential conflict of interest.

## Footnotes

1 http://www.fil.ion.ucl.ac.uk/spm

2 http://www.nitrc.org/projects/panda/

3 www.nitrc.org/projects/gretna/

4 http://www.nitrc.org/projects/bnv/

5 http://www.csie.ntu.edu.tw/~cjlin/libsvm/
