# Supplementary material for: Progressive Brain Degeneration From Subjective Cognitive Decline to Amnestic Mild Cognitive Impairment: Evidence From Large-Scale Anatomical Connection Classification Analysis
Source: Front Aging Neurosci. 2021 Jul 12;13:687530. doi: 10.3389/fnagi.2021.687530 (PMC8312851; doi:10.3389/fnagi.2021.687530)
Supplement: Supplementary file 1 [file Data_Sheet_1.PDF]

Progressive brain degeneration from subjective cognitive decline to amnestic mild cognitive impairment: evidence from large-scale anatomical connection classification analysis

Supplementary Material

Table S1 Between-group differences in network properties at different thresholds (network-level)

FN threshold	Groups	Eglobal	Elocal	CC	PL
1	HC	11.06±2.64	15.47±3.33	0.02±0.01	0.10±0.02
	SCD	9.61±2.17	14.17±2.99	0.02±0.01	0.11±0.03
	aMCI	7.97±2.16	12.44±2.90	0.02±0.01	0.13±0.04
2	HC	10.63±2.64	15.96±3.51	0.02±0.01	0.10±0.02
	SCD	9.61±2.17	14.36±3.17	0.02±0.01	0.11±0.03
	aMCI	7.97±2.16	12.69±3.16	0.03±0.01	0.13±0.04
3	HC	10.63±2.64	16.29±3.86	0.03±0.01	0.10±0.02
	SCD	9.61±2.17	14.49±3.27	0.02±0.01	0.11±0.03
	aMCI	7.96±2.17	12.77±3.34	0.03±0.01	0.14±0.04
4	HC	10.63±2.64	16.43±3.94	0.03±0.01	0.10±0.02
	SCD	9.60±2.18	14.67±3.50	0.03±0.01	0.11±0.03
	aMCI	7.94±2.18	12.92±3.65	0.03±0.01	0.14±0.04
5	HC	10.63±2.64	16.45±4.00	0.03±0.01	0.10±0.02
	SCD	9.60±2.19	14.56±3.72	0.03±0.01	0.11±0.03
	aMCI	7.91±2.21	12.67±3.79	0.03±0.01	0.14±0.04
10	HC	10.47±2.67	15.36±4.56	0.03±0.01	0.10±0.02
	SCD	9.35±2.26	12.90±4.50	0.03±0.01	0.11±0.03
	aMCI	7.51±2.38	11.37±4.50	0.03±0.01	0.15±0.06

The green part of the table is significantly different from the red part, while the unlabeled color part is not significantly different from any other group.

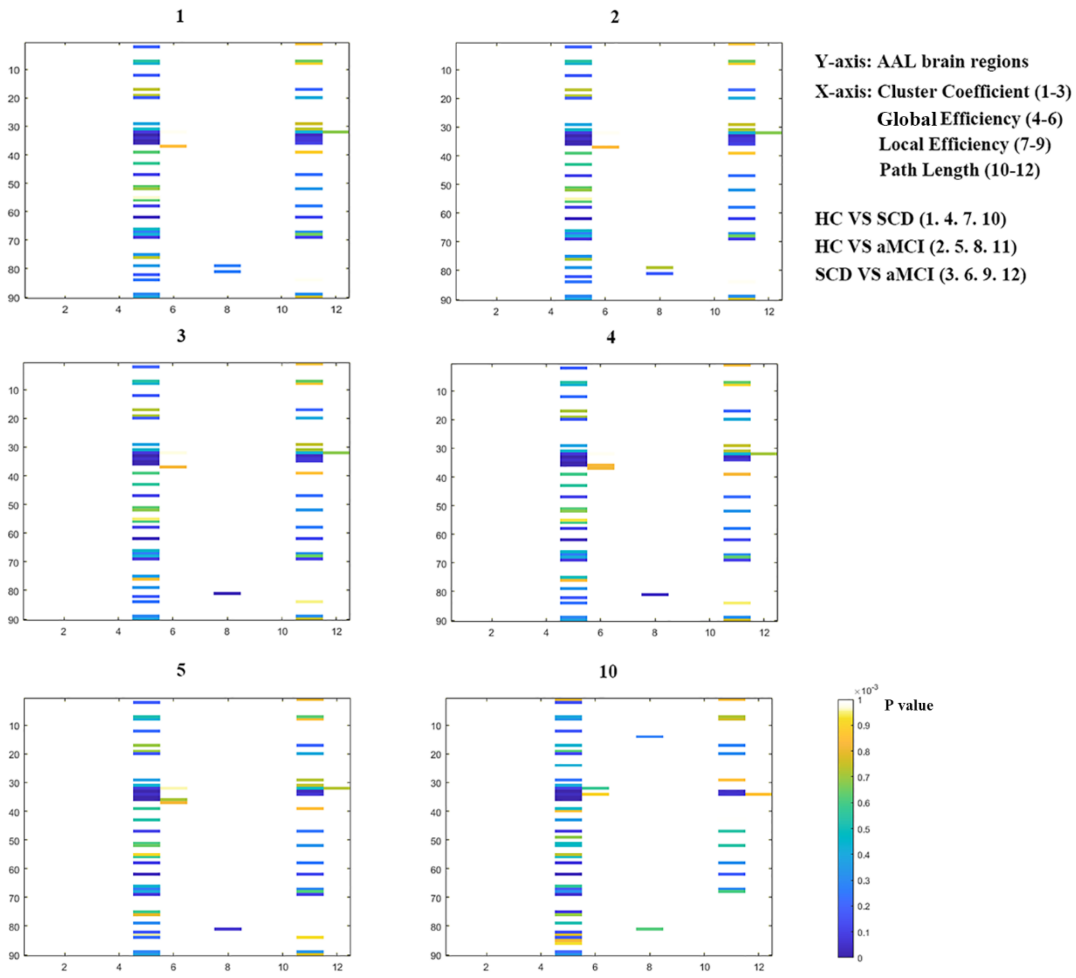

Figure S1. Between-group differences in network properties at different thresholds (at the nodal level). Y-axis of each figure indicates 90 brain regions, x-axis represents different group contrasts on different indexes at the nodal level. More specifically, 1 (cluster coefficient), 4(global efficiency), 6 (local efficiency), 10 (path length) represents contrasts between HC and SCD; 2(cluster coefficient), 5(global efficiency), 8(local efficiency), 11 (path length) represent contrasts between HC and aMCI; 3(cluster coefficient), 6(global efficiency), 9(local efficiency), 12(path length) represent contrast between SCD and aMCI. Numbers above figures represent different FN thresholds.

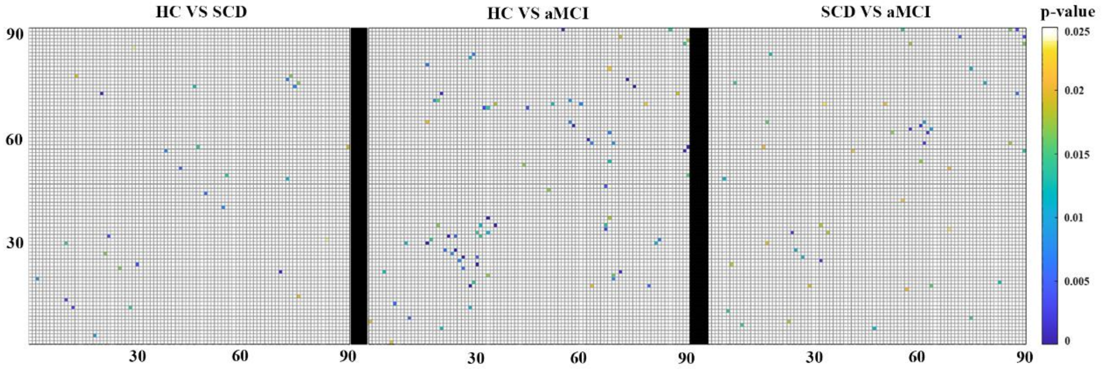

Figure S2 The between-group differences of structural connectivity. Y-axis and X-axis of each figure indicates 90 brain regions. The value in each cell represents p value of group contrast on FN of each white matter across groups.
